# The Development and Aging of the Magnocellular and Parvocellular Visual Pathways as Indicated by VEP Recordings between 5 and 84 Years of Age

**DOI:** 10.3390/vision1010007

**Published:** 2016-10-17

**Authors:** György Benedek, Gyöngyi Horváth, Szabolcs Kéri, Gábor Braunitzer, Márta Janáky

**Affiliations:** 1 Department of Physiology, Faculty of Medicine, University of Szeged, Dóm tér 10, 6720 Szeged, Hungary; 2 Laboratory for Perception & Cognition and Clinical Neuroscience, Nyírő Gyula Hospital, Lehel utca 59, 1135 Budapest, Hungary; 3 Department of Ophthalmology, Faculty of Medicine, University of Szeged, Korányi fasor 10-11, 6720 Szeged, Hungary

**Keywords:** pattern-reversal VEP, development, aging, magnocellular, parvocellular

## Abstract

It is well known that pattern reversal visual evoked potentials (VEPs) are age-sensitive. Through the use of this technique, it is possible to assess both of the major visual pathways (i.e., the magnocellular and parvocellular ones) in terms of function and development. What developmental path these pathways follow, and if they develop/age in parallel across the human lifespan is a matter of ongoing debate, yet, only a few VEP studies have dealt with this issue. This cross-sectional study examined a sample of 115 healthy volunteers aged 5 to 84 years. Beyond the standard checkerboard pattern reversal stimulation at 97% contrast, we recorded pattern-reversal VEPs at 6% contrast to selectively stimulate the M pathway and isoluminant red and green checkerboard stimulation was also used to selectively stimulate the P pathway. Our results do not support the developmental advantage of any of the pathways. The development of both pathways appear to take a remarkably long time (well into the 30s), and the signs of aging become marked over 50 years of age, especially in the case of the magnocellular pathway.

## 1. Introduction

Human visual evoked potentials (VEPs) are used widely in clinical practice and experimental work. Evaluation of VEPs assists in an understanding of physiological and pathophysiological processes in the eye and the brain. The technique has several advantages: VEPs are easy to record, and the technique is non-invasive. Importantly, a significant body of knowledge has accumulated during the last 60 years regarding the interpretation of the recordings.

Two major, parallel retinocortical pathways convey the bulk of visual information in the human central nervous system: the magnocellular (M) and the parvocellular (P) pathways. The two pathways are anatomically distinct, and are also distinguished by their response properties to simple visual stimuli, as well as by their functional role.

The M pathway originates in the parasol ganglion cells of the retina. These cells possess large receptive fields, are highly contrast sensitive, characterized by fast axonal conduction, and have a preference for low spatial and high temporal frequencies. The associated pathway is considered to be color-blind, and carries information luminance contrast, low spatial frequencies, high temporal frequencies, and both real and illusory motion [1,2,3]. The P pathway has its origins in the midget ganglion cells of the retina. Functionally, the ‘midget’ cells are the opposites of the parasols. Their receptive fields are small, their contrast sensitivity is low, they are characterized by slow axonal conduction, and they prefer high spatial and low temporal frequencies. Furthermore, the pathway arising from them carries color information, which is not characteristic of the M pathway [4,5]. Both major pathways pass through the lateral geniculate nucleus (LGN), and synaptize in different layers of the primary visual cortex to become the dorsal (M) and ventral (P) streams [6].

The temporal development of the two major pathways is still a matter of debate, especially because it is mostly studied through indirect methods that examine functions assumed to be linked to either of these pathways. Studies that concentrated mostly on the first two years of life emphasized a delay in the development of the parvocellular pathway [7,8]. Parrish et al. [9] investigated this question by comparing the development of form and motion perception in school-age children, and found no difference. Crewther et al. [10] studied low-contrast flicker sensitivity in school-age children and concluded that the development of the magnocellular pathway was delayed. Gordon and McCulloch [11] found evidence for ongoing parvocellular maturation as late as 11 years of age. Klaver et al. [12] provided imaging evidence for the delayed development of the M-pathway in the dorsal stream including the parietal lobule.

Through the use of the evoked potential technique, it is possible to assess both of the major visual pathways in parallel, in terms of function and development. Pattern reversal VEPs recorded in the standard, way at 97% achromatic contrast, are mainly of parvocellular origin. It has been demonstrated that VEP is primarily a reflection of activity originating in the central 2–6 degrees of the visual field [13]. This corresponds to the central retina [14], where the vast majority (up to 95%) of the receptors are connected to midget ganglion cells, giving rise to the parvocellular pathway [15]. Recording responses of magnocellular origin requires low contrast stimulation [16].

Pattern reversal VEPs change with age. Evoked potentials recorded from children up to five years of age [17,18,19], schoolchildren [20,21,22], adolescents [23], and the elderly [24] were analyzed in separate studies. Tobimatsu et al. investigated VEPs between 19 and 84 years of age [25], and Sokol et al. between 13 and 82 years of age [26], but comprehensive studies embracing the entire life span (i.e., describing a cross-section of the entire population) are scarce [18,27,28,29].

There is no agreement on the duration of the developmental periods in vision. Visual acuity, stereopsis, and contrast sensitivity emerge and improve dramatically within the first six months after birth. It was originally reported that basic visual functions reach adult levels within a few months (e.g., contrast, motion and orientation sensitivity) or in the first few years (grating acuity, binocularity) of postnatal life [30]. In contrast, other studies described gradual improvement in contrast sensitivity [31,32], contour integration [33] or contrast from motion up to the adolescence [34].

P100 latency seems to be the most sensitive indicator of development and aging. There is evidence to suggest that it increases until the 20s [17,19,35], but it must be added that the data of Allison et al. [20] clearly show the longest latencies between 30 and 50 years of age, similarly to the findings of Tobimatsu et al. [25]. Dustman and colleagues found the longest latencies at 29 years of age [36].

Most visual functions, both elementary and higher, significantly deteriorate with age. This has been shown in connection with motion processing [37,38], face and object recognition [39], perception of biological motion [40] and visual attention [41], to give only a few examples. Aging of the optical media [42], thinning of the retinal nerve fiber layer [43] and widespread retinal ganglion cell loss during senescence [44,45] all add up to the observed deterioration.

The effect of aging on visual evoked potentials raised interest already in the 1970s, both in animal models and human studies [46,47,48]. Justino et al. [24] found no difference between VEPs evoked by magnocellular- and parvocellular-specific stimuli. Unfortunately, that study involved subjects only 75 years of age or older, therefore, it does not allow conclusions regarding the process of aging per se.

Contrast sensitivity has often been used to address the question of the effect of aging on the two main visual pathways in both human and animal studies [48,49,50,51]. Schefrin et al. [52] found weakening scotopic contrast sensitivity at low spatial frequencies in old age. The authors suggested that the underlying cause might be the aging of the magnocellular pathway.

Given the variability of VEPs with age, and the relative lack of such comprehensive developmental and aging studies, this study targeted a population ranging from 5 to 84 years of age. The study aimed at the determination of the development and aging of VEP in this age range as reflected by the changes in the latencies and amplitudes of the main VEP components, N70, P100, and N135. We also wished to separate the major pathways so as to determine any developmental advantage. We therefore followed the approach of Schechter et al. [16] and beyond the standard checkerboard pattern reversal stimulation at 97% contrast, recorded pattern-reversal VEPs at 6% contrast to separate the M pathway; also with isoluminant red and green checkerboard to separate the P pathway. Our hypothesis was that the different parameters would show a different developmental pattern under different conditions. Based on the literature, we hypothesized that the P100 component would be the most sensitive to age, and that its development would reach its peak at 20 to 30 years of age.

## 2. Results

**Figure 1:**
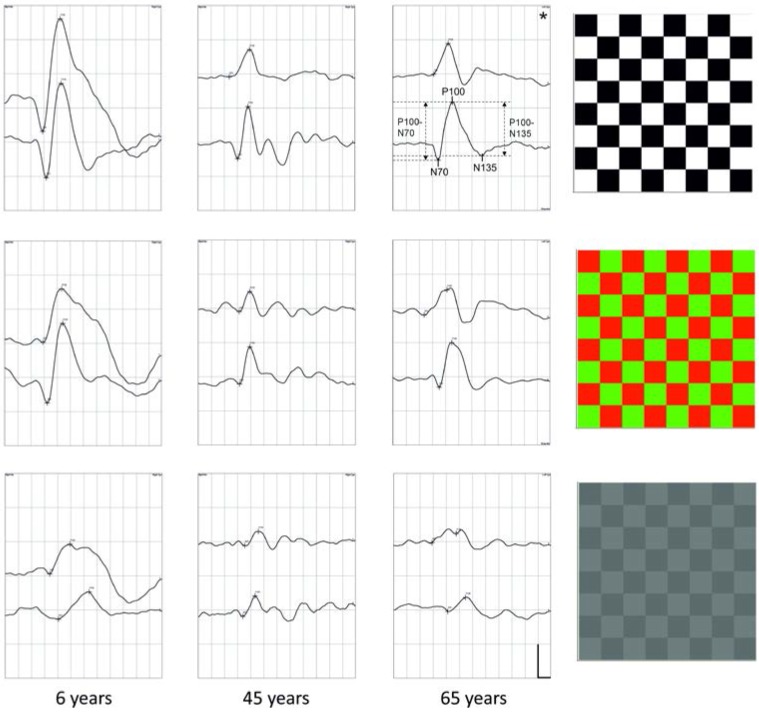
Typical averaged waveforms in response to the three types of stimulation from three randomly chosen ages. Top: 97% achromatic; middle: 97% red-green; bottom: 6% achromatic. Waveform arrangement within the recordings: top- 60′, bottom- 15′. The analyzed peaks and amplitudes are indicated in the recording marked with an asterisk. Calibration: abscissa: 25 ms/div (time); ordinate: 10 µV/div (amplitude).

The typical averaged waveforms of the three types of stimulation in different age groups are presented in Figure 1. As expected, the morphology of the waveforms differs as a function of stimulus type. The highest amplitude and shortest latency responses were evoked by high-contrast stimulation, regardless of age.

**Figure 2:**
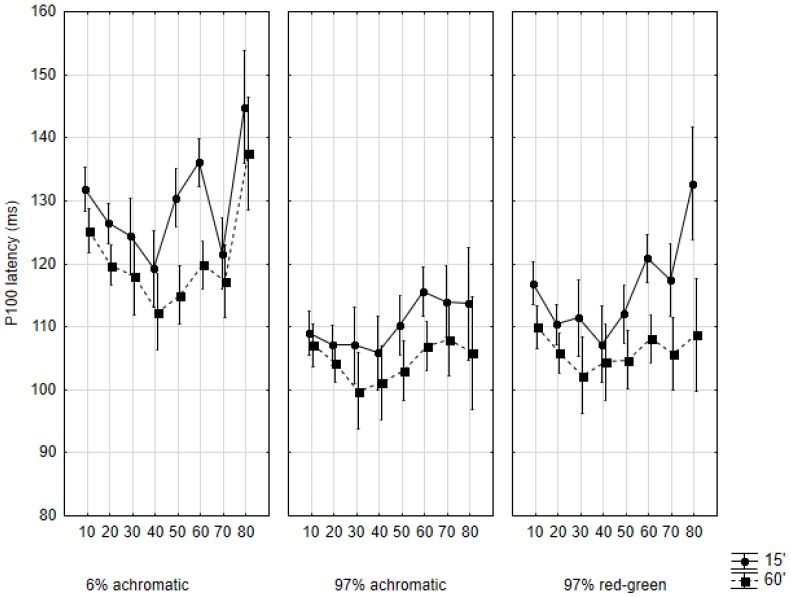
Mean P100 latencies in the studied age cohorts according to the three main conditions and by the two subconditions. Data are shown as mean ± 95% CI. The abscissa indicates the cohorts as follows: 10: 10–19 years of age, 20: 20–29 years of age, and so on.

In general, the most remarkable phenomenon identified was the protracted development of the responses. This was especially clear through the latency of the P100 peak (in all examined conditions, see Figure 2). Furthermore, across all conditions, the responses to 15′ stimulation were of higher amplitude and longer latency than those to 60′ stimulation. The statistical characterization of the development of the examined response characteristics are given below, in the following order: N70 latency, P100 latency, N135 latency, the N70-P100 amplitude, and the P100-N135 amplitude. In the description below, latency and amplitude values are given as means.

### 2.1. N70 Latency

**Figure 3:**
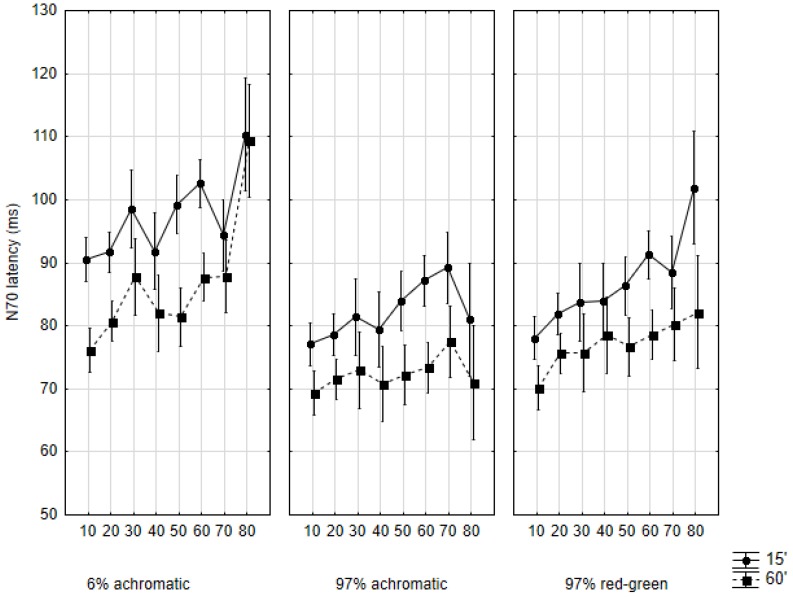
Mean N70 latencies in the studied age cohorts according to the three main conditions and by the two subconditions. Data are shown as mean ± 95% CI. The abscissa indicates the cohorts as follows: 10: 10–19 years of age, 20: 20–29 years of age, and so on.

As for the latency of the N70 response (Figure 3), the factorial ANOVA indicated a significant effect of stimulation contrast (*F*(2594) = 44.81, *p* < 0.001), stimulus size (*F*(1594) = 89.79, *p* < 0.001), cohort (*F*(7594) = 16.29, *p* < 0.001), and sex (*F*(1594) = 33.52, *p* < 0.001). The interaction of these factors was not significant (*F*(14,642) = 1.01, *p* = 0.43).

In the 6% contrast achromatic condition, the latency of N70 gets longer up to the third decade of life. In the third decade there appears to be a breaking point, the latency shortens again, and from the fourth or fifth decades it begins to get longer again. This tendency steadily continues up to the eighth decade. The development of N70 latency follows this course, regardless of the applied stimulus size. The exact latency values, though, differ by stimulus size. The latency of the N70 peak evoked by 60′ checksize starts at 75.52 (SD: 10.54) ms in the first decade of life and increases up to 111.00 (SD: 37.30) ms by the eighth decade. According to the post-hoc analysis, the difference between the latency values of the first and eighth decades is highly significant (*p* < 0.001). As for the latency values observed upon 15′ stimulation, these start at 90.10 (SD: 15.71) ms in the first decade of life, and by the eighth decade, 117.00 (SD: 38.65) ms is reached. While the pattern is the same as that observed with 60′ stimulation, and the overall difference between the first and eighth decades is significant (*p* < 0.05), 15′ latency values show variation in a narrower range (~30 ms) than 60′ latencies do (~40–50 ms) across the cohorts. For instance, the difference between the first and third decades is not significant, while in the 60′ condition, a massive difference is seen (*p* < 0.001), indicating a big developmental leap.

In the 97% achromatic condition the developmental plots are quite flat, and while these are not completely flat lines, the latency values are quite similar across the cohorts, regardless of stimulus size. The post-hoc analysis found no significant difference between any of the cohorts with either stimulus size. Similarly to the 6% condition, the 60′ latencies are shorter than 15′ latencies in general (starting at 69.31 (SD: 4.52) ms in the first decade, peaking at 77.27 (SD: 6.51) ms and falling back to 73.12 (SD: 11.69) ms again in the eighth decade), but both developmental plots stay in a similarly narrow range.

In the 97% chromatic condition we found an almost steadily increasing latency across the cohorts, regardless of stimulus size. No breaking point was evident. The difference between the first and eighth decades is significant for both 60′ and 15′ (*p* < 0.01 and *p* < 0.001, respectively). The developmental plots run parallel, and both cross an approximately 20 ms range. As before, 15′ latencies are longer (78 ms to 101 ms for the entire course of development) than 60′ latencies (70.17 (SD: 7.95) ms to 86.88 (SD: 24.25) ms).

### 2.2. P100 Latency

Similarly to the latency of the N70 response, the factorial ANOVA indicated a significant effect of stimulation contrast (*F*(2594) = 70.54, *p* < 0.001), stimulus size (*F*(1594) = 45.00, *p* < 0.001), cohort (*F*(7594) = 10.64, *p* < 0.001), and sex (*F*(1594) = 15.85, *p* < 0.001), but the interaction of these factors was not significant (*F*(14,594) = 0.63, *p* = 0.84). A common feature of the three main conditions is that the plots that belongs to the 60′ sub-condition follows a strikingly similar path (regardless of whether the changes are significant), while those belonging to the 15′ sub-condition do not show this similarity (Figure 2).

In the 6% contrast achromatic condition, the latency values follow a very similar developmental pattern in both the 15′ and 60′ subconditions (Figure 2). In both cases, a roughly U- or V-shaped plot is seen (less regular in the 15′ subcondition). Beginning at 130.73 (SD: 18.01) ms (15′) and 124.88 (SD: 14.92) ms (60′), the latency of the response in both sub-conditions steadily decreases up to the fourth decade, when it reaches 119.23 (SD: 12.75) ms (15′) and 111.81 (SD: 8.33) ms (60′). Compared to the baseline, the difference is significant in both cases at the level *p* < 0.01. Beyond the fourth decade, the latency begins to increase again, and steadily does so until it finally reaches 150.00 (SD: 35.12) ms (15′) and 139.13 (SD: 35.23) ms (60′) by the 80s. The difference, again, is significant at *p* < 0.01 for both sub-conditions. In summary, the development of the latency of the P100 responses in both the 15′ and 60′ sub-conditions may be characterized as consisting of a period of significant decrease (ages 10 to 40) and then a period of significant increase (ages 40 to 80).

Similarly to the situation described in connection with the N70 response, P100 latencies in the 97% contrast achromatic condition vary in a narrow range in all cohorts (~20 ms). While there is obviously some variability in this parameter across the cohorts, the post-hoc analysis found no significant difference between any of the cohorts with either stimulus size.

An interesting finding in the 97% contrast red-green condition is that while the latency of the 60′ responses shows almost no variability (it remains within the 100–110 ms window throughout), the 15′ responses show a marked aging effect, quite similar to that described in the 6% achromatic condition: there is a latency decrease up to the fourth decade (116.85 (SD: 9.98) ms to 107.63 (SD: 5.36) ms, *p* = 0.054), and from then point on, a steady increase is seen (up to 134.13 (SD: 8.33) ms, *p* < 0.01).

### 2.3. N135 Latency

The factorial ANOVA indicated a significant effect of stimulation contrast (*F*(2571) = 24.75, *p* < 0.001), stimulus size (*F*(1571) = 16.16, *p* < 0.001), cohort (*F*(7571) = 19.55, *p* < 0.001), and sex (*F*(1571) = 8.47, *p* < 0.001), but the interaction of these factors was not significant (*F*(14,571) = 70.54, *p* = 0.97).

**Figure 4:**
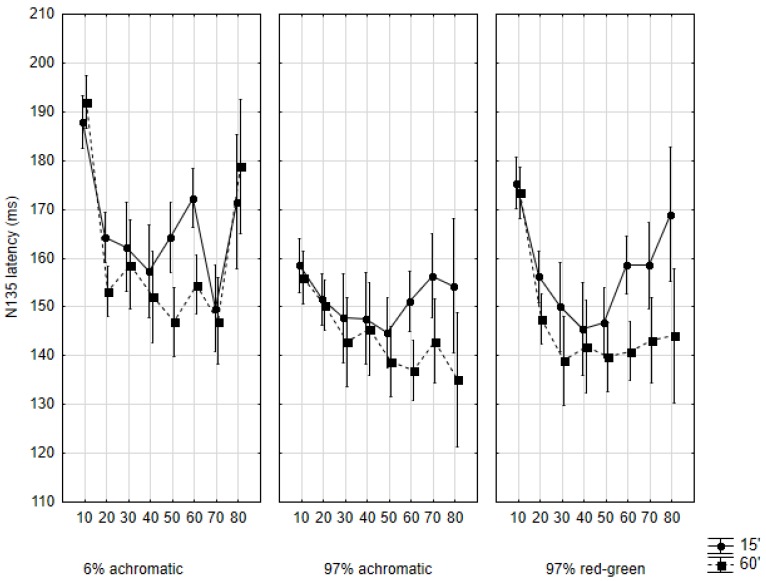
Mean N135 latencies in the studied age cohorts according to the three main conditions and by the two subconditions. Data are shown as mean ± 95% CI. The abscissa indicates the cohorts as follows: 10: 10–19 years of age, 20: 20–29 years of age, and so on.

The developmental course of this parameter showed numerous similarities with what was described under P100 (Figure 4). For instance, in the 6% achromatic condition almost exactly the same U-shaped plots can be observed as with P100 latency in the same condition (Figure 3). The only difference is that the shortest latencies for 60′ stimuli can be measured in the fifth decade of life, not in the fourth, and in the case of 15′ stimuli, even later, in the 70s. The differences are significant in both cases. As for 15′: 186.39 (SD: 30.19) ms to 150.90 (SD: 19.11) ms (teens to 70s, *p* < 0.01), then up to 176.38 (SD: 37.48) ms in the 80s (*p* < 0.01). 60′: 186.39 (SD: 30.19) ms to 150.90 (SD: 19.11) ms (teens to seventies, *p* < 0.01), then up to 176.38 (SD: 37.48) ms in the 80s (*p* < 0.01). In the 97% achromatic condition, response latencies to 15′ did not show significant variability across the cohorts. The latency values varied within an approximately 15 ms window. In contrast, when 60′ stimulation was used, the response latencies showed a steady decrease during the eight studied decades (155.48 (SD: 22.15) ms to 130.90 (SD: 21.32) ms, *p* < 0.005).

In the 97% chromatic condition, the developmental plots of response latencies exhibited the same divergence as seen with the P100 responses, that is, 15′ response latencies followed the U-shaped path (like in the 6% achromatic condition), while the 60′ latencies reached a floor in the third decade, and from that point on they did not change significantly. In the latter case, the initial mean was 173.50 (SD: 27.30) ms, which decreased to 139.06 (SD: 10.72) ms by the third decade, but this was not significant (*p* = 0.76). As for the 15′ latencies, these started at 175.33 (SD: 18.45) ms in the first decade to reach the bottom of 145.69 (SD: 9.42) ms in the fourth decade, and then to ascend to 169.50 (SD: 9.14) ms in the eight decade. These changes did not turn out to be significant either.

### 2.4. Amplitudes

**Figure 5:**
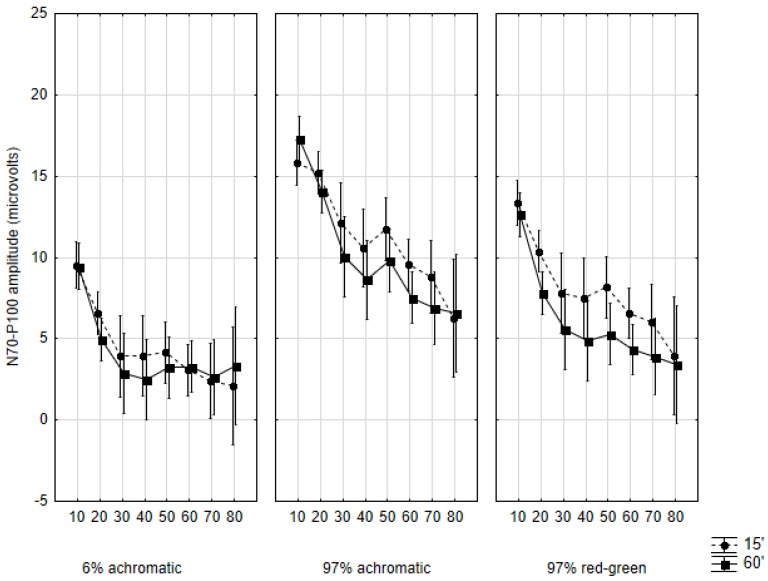
Mean N70/P100 amplitudes in the studied age cohorts according to the three main conditions and by the two subconditions. Data are shown as mean ± 95% CI. The abscissa indicates the cohorts as follows: 10: 10–19 years of age, 20: 20–29 years of age, and so on.

**Figure 6:**
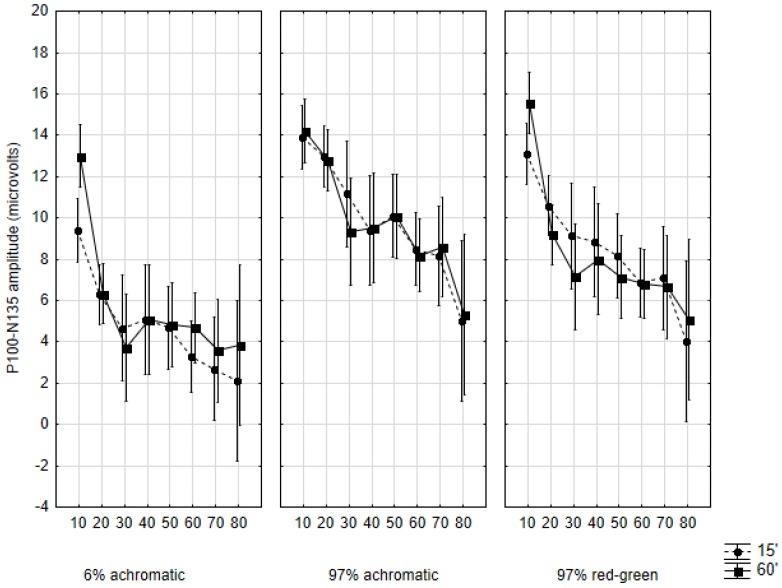
Mean P100/N135 amplitudes in the studied age cohorts according to the three main conditions and by the two subconditions. Data are shown as mean ± 95% CI. The abscissa indicates the cohorts as follows: 10: 10–19 years of age, 20: 20–29 years of age, and so on.

The developmental plots for the N70/P100 and P100/N135 amplitudes are shown in Figure 5 and Figure 6. Apart from minute differences, these plots are uniform across the conditions and sub-conditions: the amplitudes show a steady decrease from the first to the eighth decade. A significant (*p* < 0.001) linear decay tendency was found for both amplitudes in all conditions. However, unlike with response latencies, no unique developmental patterns were observed with amplitudes, therefore we forgo their detailed description.

## 3. Discussion

So far, only a few attempts have been made to describe the VEP responses as a function of stimulation across the entire lifespan. Crognale [18] published a thorough study on the development of chromatic evoked potentials during the entire lifespan, from the age of 1 week up to 90 years. According to his results, the latency of the major negative component reached the adult-like minimum at about 17–18 years of age. Throughout the rest of the life span, the latency of this component steadily increased and the amplitudes slightly decreased. Tobimatsu et al. [25] compared responses recorded at 10% and 85% levels of contrast and varying luminance. Schechter et al. [16] compared VEPs recorded with chromatic and achromatic stimulation in schizophrenic patients.

In this study, we sought to map the developmental course of the three main VEP components—as a function of age, sex, and the type of stimulation—in order to gain a better insight into the development of the magnocellular and parvocellular pathways.

First of all, as hypothesized, our findings corroborate the literature regarding P100 latency being apparently the most sensitive to age [17,19,20,35,36,46,47]. According to our findings, P100 latency decreases up to the third decade of life (development), and then shows a monotonic increase again (aging). This was especially well seen in the 6% contrast achromatic condition.

As for the P100 amplitudes, such an agreement in the literature is lacking. In a number of studies, these were found to be higher in childhood and over 60 years of age than in the period in between [25,28], but there is also evidence to suggest that aging has no effect on this parameter whatsoever [47,53]. We observed an almost monotonic decrease of this parameter throughout the studied period.

As for the waveform of the P100 response, we observed double peaked P100 components in every condition quite frequently, and their numbers increased with the subjects’ age. In the clinical practice, this phenomenon is often considered to be a sign of demyelinization [54], but Stothart et al. [55] pointed out that the finding can be perfectly normal in healthy aging as well.

The development of the latency of the N135 response was remarkably similar to that of P100 latency across all conditions. This is possibly secondary to the changes in P100 amplitude (i.e., under healthy conditions the period between N70 and P100 is not highly variable, which means that the latency of N135 “follows” the latency of P100). The N70 latencies showed a quasi monotonic increase with age. This observation may reflect the decreasing conduction velocity along the optic nerve [56], although there is no consensus regarding when that decreasing tendency sets on. P100 latency and the amplitude of N135 (the amplitude between P100 and N135) showed the most conspicuous differences between high contrast and low contrast stimulation. This could be in connection with the finding that N135 is generated in the extrastriate areas [57,58].

A further finding of importance in connection with the P100 and N135 responses was that the development of their latency was protracted. Regardless of stimulation, these latencies did not reach their minimum before the third decade of life. This was somewhat longer than we hypothesized, but the finding itself was not unexpected at all: previous studies already reported on the long development of this parameter [20,25,36].

As for the developmental advantage of either of the major pathways, our data suggest no such advantage, or even if there is an advantage, it will not show in the examined parameters. We hypothesized that the different parameters would show a different developmental pattern under different conditions. This hypothesis failed. For any given VEP parameter evoked with either of the two check sizes, the developmental plots were more or less identical, regardless of contrast and color.

A general finding was that sex proved to be a significant determining factor of all components and in all conditions. This came as no surprise, as sex differences in the VEP responses are known [59,60]. Still, as 76 percent of our sample were females, this finding rather reflects the sex imbalance of the sample than a unique sex effect.

## 4. Material and Methods

Altogether 115 subjects participated in the study (87 females, 28 males, age: 5 to 84 years). The subjects were volunteers recruited from among the students of the University of Szeged, neighboring schools and kindergartens and from among the patients of the Department of Ophthalmology who, after ophthalmological examinations, were declared healthy. Negative ophthalmological and neurological status was inclusion criteria. Therefore, before the VEP recordings, all subjects were screened for ophthalmological alterations, and negative neurological status was verified from patient records. In addition, only volunteers with a 20/20 vision were eligible for the study (with or without correction).

The study protocol conformed to the tenets of the Declaration of Helsinki in all respects, and was approved by the Ethics Committee for Human Medical Biological Research at the University of Szeged. Prior to the VEP recordings, all subjects (and their parents if the subject was under 18 years of age) were informed about the aims and procedures of the research, both in written and oral form. Care was taken to provide children with information appropriate to their age. Written informed consent was obtained from all participants, and in the case of children, by their parent or guardian. All of the procedures to record VEPs were performed in accordance with the recommendations and standards of the International Society for Clinical Electrophysiology of Vision (ISCEV) [7].

The RETIport 32 software of a Roland device (Roland Consult, Wiesbaden, Germany) was used for both stimulation and recording. Checkerboard patterns with checksizes of 15′ (0.25°) and 60′ (1°) of visual angle served as stimuli at a reversal rate of 0.9 Hz. The viewing distance was 33 cm, and the stimulus display subtended an area of 12° (vertical) by 16° (horizontal). The luminance of the stimulation screen was set at 100 cd/m^2^.

Pattern VEPs were recorded monocularly from both eyes, with corrected refraction, without pupil dilation, by means of gold-cup electrodes. The place of the recording electrode was the Oz site, the reference electrode was fastened at Fz, and the ground electrode on the forehead. The band pass filter was set to 1–100 Hz. One hundred responses were averaged.

Three conditions were presented; a 97% achromatic contrast condition, a 6% achromatic contrast condition, and a chromatic contrast condition. The chromatic condition was presented at isoluminance to null the response of the M system. Isoluminance refers to the condition when there is no difference in luminance across a spatial pattern that is defined in terms of chromatic contrast. To determine the isoluminant point for each participant, the electrophysiological technique of Zemon and Gordon [61] was used, and isoluminance was estimated through manipulation of the ratio of red and green guns of the RGB display monitor. We can not exclude, however, some contamination of the responses from change of luminance on the display. The recording was performed at room light for the 97% achromatic and chromatic conditions and at mesopic lighting (0.4 lux) for the 6% achromatic condition.

For the evaluation of the VEP responses, the N70, P100, and N135 latency times and the N70/P100 and P100/N135 amplitudes were used. Latency was measured as the distance of the given peak from time point zero. Amplitudes were measured as the distance between the N70 and the P100 peaks and between the P100 peak and the N135 peak (see Figure 1).

Averaged values from both eyes were used. The sample was divided into eight cohorts by decades (i.e., cohort 10: 10–19 years, cohort 20: 20–29 years, etc.). The number of participants in each cohort was as follows: *n*_10_: 24, *n*_20_: 27, *n*_30_: 8, *n*_40_: 8, *n*_50_: 13, *n*_60_: 20, *n*_70_: 11, *n*_80_: 4. We divided the sample into cohorts like this because we sought to find out if there is any specific breaking point in the development of the studied parameters. The data were analyzed using factorial ANOVA by checksize, contrast, cohort, and sex. Latencies and amplitudes were compared across these cohorts to characterize development. For the post-hoc comparisons, the Newman-Keuls post hoc test was used. The general level of significance was set at α = 0.05. For the analyses, STATISTICA for Windows 12.0 (Statsoft Inc., Tulsa, OK, USA) was used.

## 5. Conclusions

In general, this study supports most of the findings in the literature relating to the development and aging of VEP components. We showed that the latencies of P100 and N135 have a prolonged development that lasts until the third decade of life. It is only then that aging (latency increment) starts. The latency of N70 increased throughout the examined period, which possibly shows the aging of the optic nerve. Both studied amplitudes showed steady decrease. The characteristics of the evoked responses showed that our pathway-selective stimulation method worked, however, we were unable to demonstrate any developmental advantage of either of the two major visual pathways.
